# From Needs Assessment to Pre-Trial Specification: Participatory Co-Design of a Generative AI Chatbot (CARES-MDD) for Persons Living with Depression in Hong Kong Psychiatric Care

**DOI:** 10.3390/healthcare14203547

**Published:** 2026-10-21

**Authors:** Heidi Ka Ying Lo, Michelle Sze Yiu Yu, Paul Kin Shing Sze, Rachel Wai Kiu Wong, Michelle Chi Ting Ku, Yolanda Yui Huen Cheng, Peter Hiu Fung Ng, Wing Fai Yeung, Ka Fai Chung

**Affiliations:** 1 Department of Psychiatry, School of Clinical Medicine, LKS Faculty of Medicine, The University of Hong Kong, Hong Kong SAR, China; michyu34@hku.hk (M.S.Y.Y.); paulsze@connect.hku.hk (P.K.S.S.); rache1wk@connect.hku.hk (R.W.K.W.); u3627142@connect.hku.hk (M.C.T.K.); yc0815@connect.hku.hk (Y.Y.H.C.); kfchung@hku.hk (K.F.C.); 2 Department of Computing, The Hong Kong Polytechnic University, Hong Kong SAR, China; peter.nhf@polyu.edu.hk; 3 Department of Rehabilitation Science, The Hong Kong Polytechnic University, Hong Kong SAR, China; 4 School of Nursing, The Hong Kong Polytechnic University, Hong Kong SAR, China; jerry-wf.yeung@polyu.edu.hk

**Keywords:** generative artificial intelligence, digital mental health, young people, mental health, depression, help-seeking, mental health stigma, cognitive behavioral therapy, participatory co-design

## Abstract

**Highlights:**

**What are the main findings?**

• Among psychiatric outpatient attendees, 66.2% (CI 61.9–70.1%) reported prior use of generic AI chatbots for emotional concerns. Fear of negative judgment and burdening others were recurrent themes of barriers to sharing emotional concerns.
• “A broad psychiatric outpatient needs assessment and adult lived-experience co-design process informed CARES-MDD, a Hong Kong Cantonese, Cognitive Behavioral Therapy (CBT)-informed generative-AI conversational-agent prototype for adjunctive support in psychiatric care.”

**What are the implications of the main findings?**

• Generative AI chatbots with specified features are needed. Content analysis identified positive and negative points in users’ experience during the development process.
• This study provides a stakeholder-informed intervention specification and design parameters for a subsequent controlled evaluation of CARES-MDD.

**Abstract:**

**Background/Objectives**: The burden of major depressive disorder (MDD) calls for assessing the needs and design of innovative add-on interventions. Generative artificial-intelligence (AI) tools are increasingly used in everyday life. Evidence remains limited on how needs assessment and co-participatory design can inform AI chatbot trials for MDD. **Methods**: This study reports a mixed-methods design. Phase 1 comprised a needs assessment of 511 psychiatric outpatient attendees in Hong Kong. Phase 2 involved a participatory co-design and test-run experiences with 10 lived-experience experts with MDD and eight multidisciplinary clinical professionals. This process informed the development of CARES-MDD, a Hong Kong Cantonese, CBT-informed generative AI conversational-agent prototype intended to provide adjunctive support alongside usual psychiatric care. **Results**: In Phase 1, 66.2% reported using generic AI chatbots for emotional concerns; privacy, anticipated negative evaluation, and concern about burdening others were recurring themes in the qualitative data. Localized empathy, non-judgmental, and 24/7 accessibility were the main themes of a desired mental health generative AI chatbot. In Phase 2, CARES-MDD was co-designed and assessed against a 7-domain feedback rubric measuring perceived usefulness and acceptability. Overall, CARES-MDD received favorable feedback rubric ratings. Content analysis identified positive and negative experiences, which established parameters for future trial methodology. **Conclusions**: This study identifies needs and yields the co-design of the CARES-MDD purposed as adjunct support for MDD in psychiatric care in Hong Kong, providing the parameters to inform future controlled trials.

## 1. Introduction

Major depressive disorder (MDD) is a leading cause of disability worldwide [1], accounting for substantial disease burden, diminished functional capacity, and elevated suicide risk [2,3]. While empirically validated psychological interventions, most notably cognitive behavioral therapy (CBT), are recommended as first-line treatments, fewer than half of affected individuals receive adequate care [4,5].

Digital mental health interventions (DHIs) may increase access to information and self-management support; however, sustained engagement, cultural relevance, and integration with routine psychiatric care remain important challenges [6,7]. Critically, the overwhelming majority of mental health DHIs are developed without participatory input from clinical populations, producing tools that feel generic, robotic, and emotionally misaligned for individuals contending with severe depressive symptoms [8,9].

The emergence of generative artificial intelligence (AI) and large language models (LLMs) offers an opportunity to bridge this gap. Unlike rigid, rule-based algorithms, LLMs can generate responsive language that may be perceived as empathic or validating; however, such outputs are not equivalent to human understanding or clinician-delivered therapy and require empirical safety and efficacy evaluation [10,11,12]. When integrated with evidence-based psychotherapeutic frameworks—specifically CBT, which targets transdiagnostic cognitive and behavioral maintaining mechanisms (e.g., cognitive distortions, avoidance, and rumination [13,14])—generative conversational agents can deliver flexible, tailored micro-interventions in real time.

However, translating generative AI into a psychiatric adjunct treatment tool requires tuning, localized adaptation, and safety governance. This formative mixed-methods study aimed to identify user-defined requirements, stakeholder-identified failure modes, and governance conditions for the development of Conversational Artificial-intelligence for REducing Symptoms and REsponse for Major Depressive Disorder (CARES-MDD), which is a Hong Kong Cantonese AI-supported conversational agent for persons living with depression. This study was designed to inform a prespecified, staged validation program for CARES-MDD, including content curation, technical evaluation, safety assurance, and prospective feasibility testing before an efficacy trial. Specifically, this study sought to (1) characterize digital behavior, generic AI chatbot use, barriers to emotional disclosure, and desired chatbot features among people attending psychiatric outpatient services; (2) translate these findings, together with feedback from lived-experience experts and clinical professionals, into prioritized generative AI chatbot design recommendations; and (3) describe stakeholder perceptions of perceived usefulness and acceptability using feedback rubrics and qualitative interviews to collect their positive and negative points when testing CARES-MDD.

## 2. Materials and Methods

### 2.1. Study Design

This study employed a mixed-methods, participatory co-design framework, drawing upon established guidelines for DHIs [15,16,17]. Recognizing that the target demographic has important experience and opinions of generative AI, the investigation was structured into two interrelated phases:

**Phase 1 (Needs Assessment):** A cross-sectional quantitative and qualitative survey investigating baseline digital habits, help-seeking attitudes, self-stigma, and conversational AI acceptability/needs among adolescents and young people attending child and adolescent psychiatric outpatient services. This cohort was not restricted to individuals with MDD for real-world generalizability.

**Phase 2 (Participatory Co-Design and Test-run Evaluation):** A stakeholder co-design/feedback phase involved adult lived-experience experts (LEEs) advisers and multidisciplinary clinical professionals. The adult-only reference group was used to refine the initial prototype, identify design and governance considerations, and discuss sensitive issues relating to emotional disclosure and AI-supported mental health care. Phase 1 findings were used to identify candidate design priorities, including privacy and discretion, use pattern, low-burden access and barriers reported. Phase 2 included a workshop of LEEs to integrate the prioritized implementation ideas to co-design the generative AI chatbot (CARES-MDD) and then invited them for test-run CARES-MDD to evaluate its acceptability, perceived usefulness and perceived safety in mixed-methods evaluation for its potential for progression to a randomized controlled trial (RCT) design.

### 2.2. Participants and Recruitment Settings

**Phase 1 Cohort.** A clinical cohort of 511 outpatient psychiatric attendees was recruited from Child and Adolescent Psychiatric Outpatient Clinics at Queen Mary Hospital Service attending between 22 July and 21 August 2025 during routine clinical visits. Recruitment was conducted by trained research interns who were stationed in the clinic during selected weekday clinic sessions, according to intern availability. During staffed sessions, interns sequentially approached patients and, where applicable, their parent or guardian to introduce the study, provide study information, and invite voluntary participation. Participation or non-participation did not affect clinical assessment or treatment. The sample was a time–location convenience sample. Although recruitment was conducted sequentially among patients encountered during each staffed session, the recruitment opportunities depended on intern availability and the clinic sessions covered. The service did not operate diagnosis-specific clinics; therefore, patients attending the general child and adolescent psychiatric service during staffed sessions could be approached regardless of their presenting diagnosis. This cohort was not restricted to individuals with MDD, and depressive symptom severity was assessed using the Patient Health Questionnaire-9 (PHQ-9). Eligible participants possessed a personal smartphone with daily internet access and demonstrated the ability to read written Chinese and communicate in Cantonese. No formal a priori sample-size calculation was performed for Phase 1. As this was a formative, descriptive needs assessment, the sample size was determined pragmatically by the number of eligible patients who consented during the prespecified four-week recruitment period and staffed clinic sessions. All enrolled participants were included in the analysis.

**Phase 2 Reference Group.** The Phase 2 participatory co-design involved the interview of eighteen experts, comprising ten LEEs, alongside a multidisciplinary expert group of eight clinical professionals (two clinical psychologists, three counselors, one psychiatrist, and two psychiatric nurses). LEEs had a confirmed clinical diagnosis of major depressive disorder (MDD) based on the Diagnostic and Statistical Manual of Mental Disorders, Fifth Edition (DSM 5) [18], ascertained by case psychiatrists. This diagnostic criterion applied only to Phase 2 LEEs and did not apply to the broader Phase 1 outpatient needs-assessment cohort. The LEEs were all aged 18 years or above; restricting the reference group to legal adults was intended to ensure that participants had the developmental maturity to discuss lived experiences of depression, emotional disclosure, and digital mental health support in an interview setting. Exclusion criteria included acute suicidal risk, active psychosis, or concurrent substance use disorders. While the LEEs guided the humanistic and user-experience elements, the clinical experts provided feedback on the content and appropriateness of the interaction. Furthermore, the expert panel provided opinions on the safety features in practical use.

### 2.3. Phase 1: Needs-Assessment Measures and Data Collection

An electronic questionnaire was developed to capture quantitative metrics and qualitative insights across four primary domains:

**Online behavior and AI chatbot use:** Their daily digital screen time, generic conversational AI adoption rates, daily online interaction duration, and digital engagement frequency were asked. Depressive symptom severity was assessed using the validated Chinese version of the 9-item Patient Health Questionnaire (PHQ-9) as a clinical variable [19]. The PHQ-9 was used to quantify self-reported depressive symptom burden and to classify symptom-level groups for analysis; it was not used to establish a DSM-based diagnosis of MDD.

**Help-Seeking and Self-Stigma:** Standardized psychometric instruments measured help-seeking behaviors (Help-Seeking Scale, max score 28) and internalized mental health stigma (Self-Stigma Scale, max score 24, where higher scores denote lower stigma) from the Knowledge and Attitudes to Mental Health Scales (KAMHS) [20], which was recently validated in Hong Kong [21].

**Barriers to emotional disclosure and help-seeking within existing psychiatric care networks:** Participants also completed an open-ended item asking “What are the reasons that prevent you from sharing emotional difficulties with others?”

**Desired AI Chatbot Specifications:** To guide the system architecture and feature prioritization, a final open-ended item asked “What is the most desired feature of an AI chatbot within the existing psychiatric system?”

### 2.4. Phase 2: Co-Design Workshop, Beta-Runs, and Mixed-Methods Evaluation

To translate the needs-assessment findings into actionable generative AI chatbot development, a participatory co-design workshop was conducted with lived-experience experts (LEEs). The engagement protocol was grounded in established youth participatory action research (YPAR) principles [22] and guidelines for co-designing digital health interventions with young people [23], which used non-medicalized language, culturally relevant humor, and open body language (e.g., maintaining eye-level seating) to actively cultivate trust and rapport. Active listening techniques, such as reflective summarizing and open-ended prompting, were utilized to verify shared understandings, clarify conceptual misinterpretations, and empower LEEs to expand upon their ideas on the prioritization of the generative AI chatbot theme. This collaborative process directly informed the prioritized implementation strategies for the Conversational Artificial-intelligence for REducing Symptoms and REsponse for Major Depressive Disorder (CARES-MDD) intervention (detailed in Appendix A Table A1), bridging the gap between clinical utility and user-centered design.

Following the initial co-design workshops, the expert group was invited for multiple test-run cycles conducted over a development window. The expert reference group attended several supervised, in-person test-runs throughout this period to report any good and bad points about the CARES-MDD. At the conclusion of this iterative cycle, participants completed a feedback rubric encompassing seven study-specific domains, which were generated directly from the prioritized co-design strategies (Appendix A Table A1). The rubric was not a previously validated instrument, was not separately pilot tested, and was not intended to establish psychometric validity, formal consensus, or predefined feasibility criteria. Participants rated these domains on a 10-point visual analog scale (1 = completely inadequate, 10 = exceptional), as shown in Table 1. The seven-domain feedback rubric was designed as a joint stakeholder instrument rather than as separate patient-reported and clinician-reported measures, reflecting the co-design principle that acceptability and trial readiness should be triangulated across lived-experience and clinical-governance perspectives simultaneously, rather than evaluated as independent, potentially divergent outcomes. Given the small number of raters in each subgroup (10 LEEs; 8 clinical professionals), subgroup-stratified estimates would carry limited descriptive stability and were not considered informative at this sample size.

#### Description of the CARES-MDD Chatbot

The CARES-MDD application is a text-based, multi-threaded chatbot application developed using a large language model and designed to communicate in natural Hong Kong-style Cantonese. Its conversational content was developed by psychiatrists and clinical psychologists and reviewed by members of the clinical team with reference to evidence-informed, primarily CBT principles. To address clinical safety, CARES-MDD has a keyword-based crisis-signposting module screening for expressions associated with suicidal ideation, death wishes, self-harm, hopelessness, and possible acute risk, including terms such as “suicidal”, “death”, and “giving up”. Detection of these triggers initiates an automated safety interface,’ temporarily suspends the generation of routine chatbot content, and displays a static emergency interface with 24/7 direct-dial crisis hotlines. The research team also conducted periodic clinical review of chatbot content and system responses to identify potential safety concerns on a weekly basis. CARES-MDD was designed to support compliance with applicable data-protection requirements. Appropriate measures were implemented to protect participant information and restrict access to study data to authorized members of the research team. It was not intended to diagnose MDD, provide emergency care, replace professional assessment, or autonomously manage suicidal risk. The CARES-MDD system identity, intended use, and technical configuration, together with its safety-screening and crisis-response pathway, are summarized in Appendix A Table A2, Table A3 and Table A4.

### 2.5. Analysis

We used descriptive statistics and inferential tests to examine closed-ended questions regarding participants’ digital behaviors, help-seeking patterns, and the evaluation rubrics. Quantitative data was organized and checked in Microsoft Excel, and statistical analyses were conducted in R statistical software (version 4.3.0; R Foundation for Statistical Computing, Vienna, Austria). In Phase 1, inferential analyses were restricted to comparisons of two clinically relevant psychosocial outcomes—help-seeking score and self-stigma score—between participants with elevated versus lower depressive symptoms. Depressive symptom groups were defined using the Patient Health Questionnaire-9 (PHQ-9): participants with a PHQ-9 score of ≥10 were classified as having elevated depressive symptoms, while those with a PHQ-9 score of <**10** were classified as having lower depressive symptoms. This symptom-based grouping was used for analysis only and does not represent a DSM-based diagnosis of major depressive disorder. Between-group comparisons of continuous outcomes. Welch’s unequal variances *t*-tests were used to compare mean help-seeking and self-stigma scores between the two PHQ-9 symptom groups according to distributional and variance considerations because equal variances were not assumed. Analyses used available cases for each outcome; no missing-data imputation was performed. Continuous variables were examined for distributional characteristics before summarization. Variables with clearly right-skewed or zero-bounded distributions, including digital-engagement durations, were reported as medians with interquartile ranges (IQRs). Approximately symmetric psychometric and usability measures were summarized as means with standard deviations. Categorical variables, such as generic AI adoption rates and interaction frequencies, were reported as frequencies and percentages. Inferential analyses remained restricted to the prespecified comparisons of help-seeking and self-stigma scores between participants with elevated and lower depressive symptoms. These comparisons were conducted in R’s base function stats::t.test() with var.equal = FALSE, corresponding to Welch’s unequal-variances *t*-test. Welch’s test was selected a priori because the two symptom groups were of unequal size and equality of variances was not assumed; no Student’s equal-variance *t*-tests were used. Analyses used available cases for each outcome, and no missing-data imputation was performed. No formal adjustment for multiple comparisons was applied because the inferential analysis was restricted to two prespecified clinically relevant psychosocial outcomes rather than a broad set of exploratory tests.

For qualitative data derived from Phase 1, open-ended responses were generally brief phrases or one-to-two-sentence comments. Short-text responses were coded to categorize non-mutually exclusive recurring themes (e.g., privacy concerns, fear of judgment). To provide cultural context, frequently occurring Chinese terms within these responses were mapped and visualized using word clouds. The qualitative feedback of Phase 2 was conducted as an inductive, data-driven iterative process [24]. Due to limitations within the dataset (short phrases or sentence fragments), full thematic analysis was not appropriate [25]. Two researchers (M.S.Y.Y. and K.Y.H.L.) independently coded approximately 30% of responses using inductively generated, non-mutually exclusive descriptive codes. Multiple codes could be assigned when a response contained more than one meaning. They compared coding decisions, discussed discrepancies, and reached negotiated agreement to refine the coding framework, which was then applied to the remaining responses. No formal inter-rater reliability statistic was calculated because coding was inductive and non-mutually exclusive, and the purpose was descriptive categorization rather than validation of a fixed coding instrument. Codes were grouped into recurring categories and summarized by frequency. For Phase 2, brief spoken and written feedback on positive and negative aspects of the test-runs was analyzed using inductive descriptive content analysis. The same researchers generated codes directly from the data, discussed coding differences, and reached negotiated agreement on final categories. Related categories were grouped into broader themes, to identify prototype-refinement priorities.

### 2.6. Ethical Approvals and Consent Procedures

The assessment received institutional review board approval from the Institutional Review Board of The University of Hong Kong/Hospital Authority Hong Kong West Cluster (UW25-299). Informed consent was obtained from adult participants and their parents (for minors) prior to engagement. Participant confidentiality was secured by decoupling contact information from survey data and assigning de-identified alphanumeric study IDs.

## 3. Results

### 3.1. Phase 1 Results: Clinical Needs Assessment for Barriers and AI Chatbot Use

The cohort comprised 511 young people (mean age 14.97 ± 1.86 years; 54.0% female, 46.0% male). Analysis of online behavior revealed a significant divergence based on psychological distress levels. No patient-level information was logged for individuals who were not approached, were not eligible, or declined participation. Accordingly, no participant-versus-non-participant comparison was possible. Participants recorded a significantly high digital footprint, spending an average of 296.3 min per day online. Nearly two out of three young people (66.2%; 95% CI: 61.9–70.1%) are already familiar with and have navigated generic AI chatbots, implying the clinical population is already primed for conversational agents. While adoption is high, frequency data highlights a critical gap: a total of 56.4% of active users have only had sporadic, surface-level interactions (fewer than 10 conversations total). This suggests that, while young people are curious about AI chatbots, current generic AI chatbot tools are not providing the sustained, meaningful engagement required for psychological intervention for the clinical demographic (Table 2).

**Figure 1:**
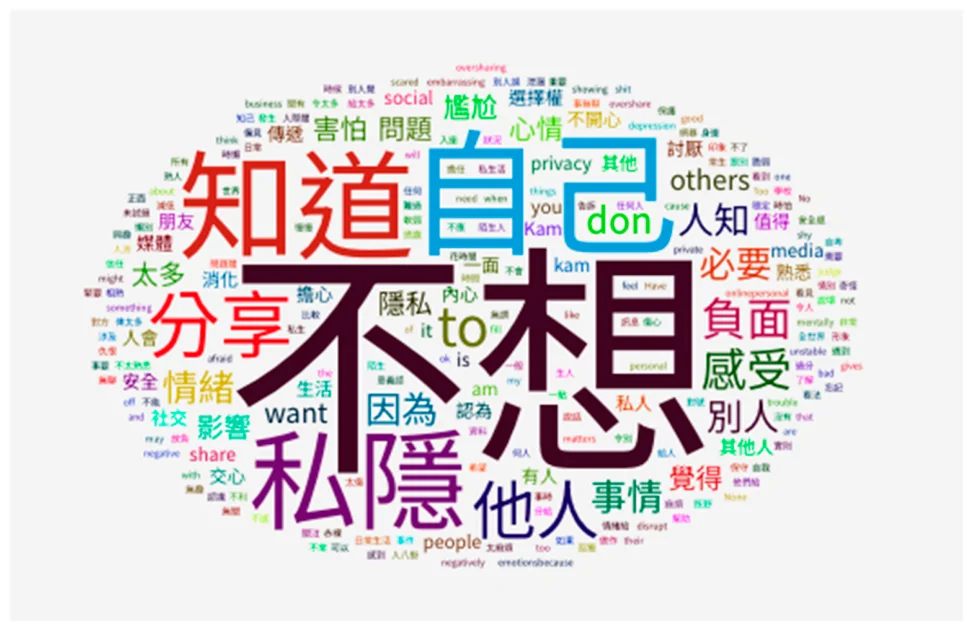
**Word cloud of frequently occurring Chinese terms in participants’ qualitative comments regarding barriers to sharing emotional difficulties with others.** Larger words represent terms that appeared more frequently in the comments. Prominent terms included “私隱” (“privacy”), “分享” (“sharing”), “感受” (“feelings”), “別人” (“others”), “負面” (“negative”), and “需要” (“need”).

Of the 511 participants, 65 (12.7%) provided additional comments in response to the question “What are the reasons that prevent you from sharing emotional difficulties with others?” Comments were generally brief, comprising a phrase or one to two sentences (total word count = 834). Iterative thematic analysis identified **Privacy concerns** (*n* = 22), characterizing emotional disclosure as “privacy” or a “personal matter” and expressing concern that their concerns can be gossiped about to others; **Fear of judgement** (*n* = 16) encompassed anticipated misunderstanding, criticism, stigma, or negative evaluation, as reflected in comments such as “other people’s views and prejudices” and “afraid of being misunderstood”; and **Avoidance of burdening others** (*n* = 8) captured concerns that disclosure might negatively affect or inconvenience others, illustrated by “do not want to affect other people’s mood” and “do not want to disturb others”. These are visualized in the word cloud in Figure 1.

**Figure 2:**
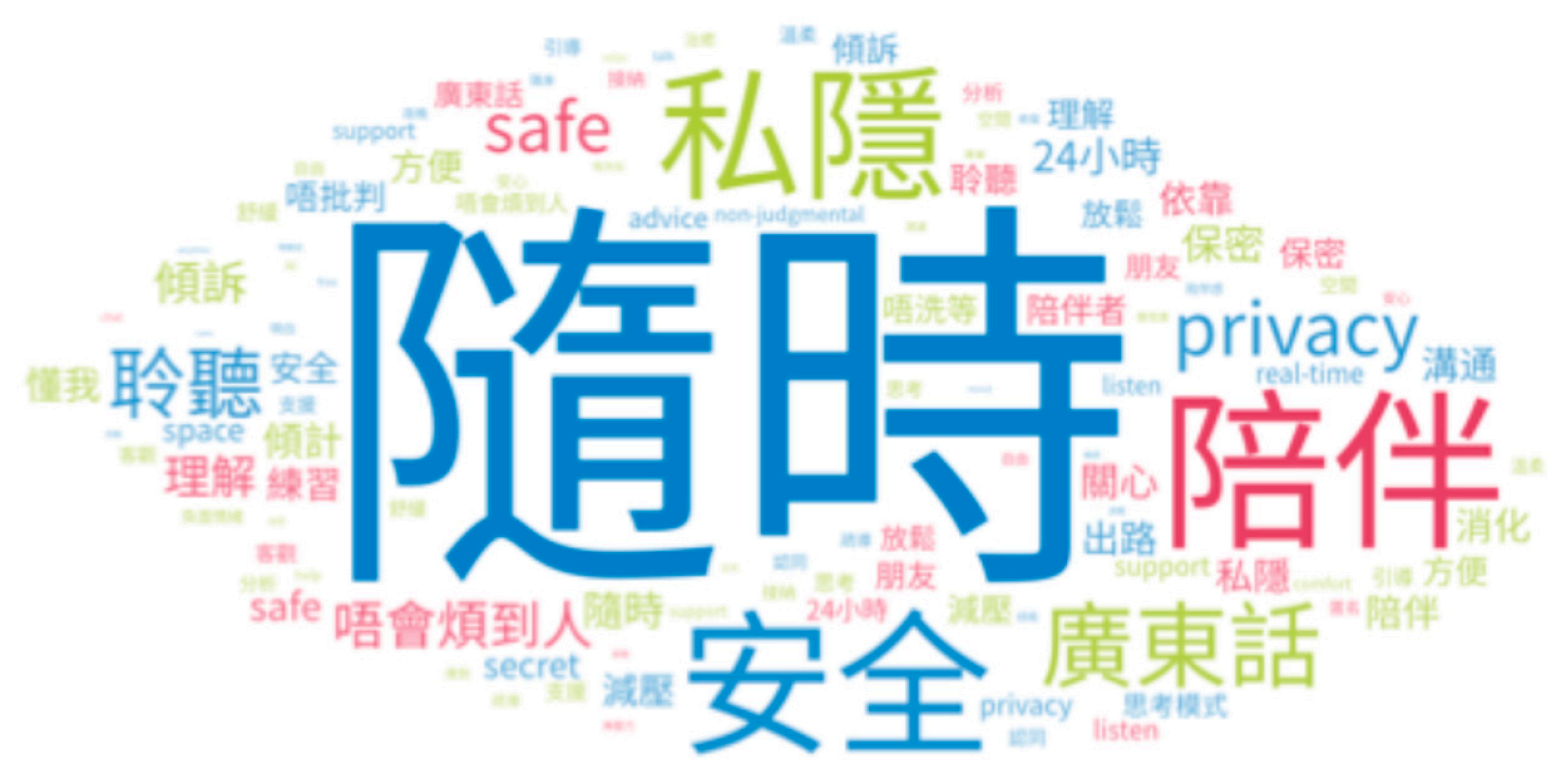
**Word cloud of frequently occurring Chinese terms in participants’ qualitative comments regarding the most desired features of CARES-MDD.** Larger words represent terms that appeared more frequently in the comments. Prominent terms included “隨時” (“anytime”), “安全” (“safe”), “陪伴” (“accompany”), “廣東話” (“Cantonese”), “私隱” (“privacy”) and “聆聽” (“listen”).

A total of 42 (8.2%) provided additional open-ended comments in response to the question “What is the most desired feature of an AI chatbot within the existing psychiatric system?” Comments were generally brief, comprising short phrases or single sentences (total word count = 586). Iterative thematic analysis identified four non-mutually exclusive desired features. The category most frequently identified among the 42 respondents who provided comments was **24/7 immediate accessibility** (*n* = 18), with participants valuing on-demand emotional support between formal clinical appointments (e.g., “always available” and “immediate reply when I cannot sleep”). **Privacy and non-judgmental space** (*n* = 12) reflected the preference to share vulnerable emotions without fear of human evaluation, characterized by comments like “no human judgment” and “completely confidential”. **Localized empathy** (*n* = 8) captured the desire for naturalistic, Hong Kong-style Cantonese interactions that feel culturally resonant rather than robotic (e.g., “sounds like a local friend”). Finally, **practical psychological guidance** (*n* = 4) referred to the need for actionable coping strategies, exemplified by “helps me reframe my negative thoughts”. These are visualized in the word cloud in Figure 2.

### 3.2. Phase 2 Results: Co-Participatory Design and Expert Evaluation

**Figure 3:**
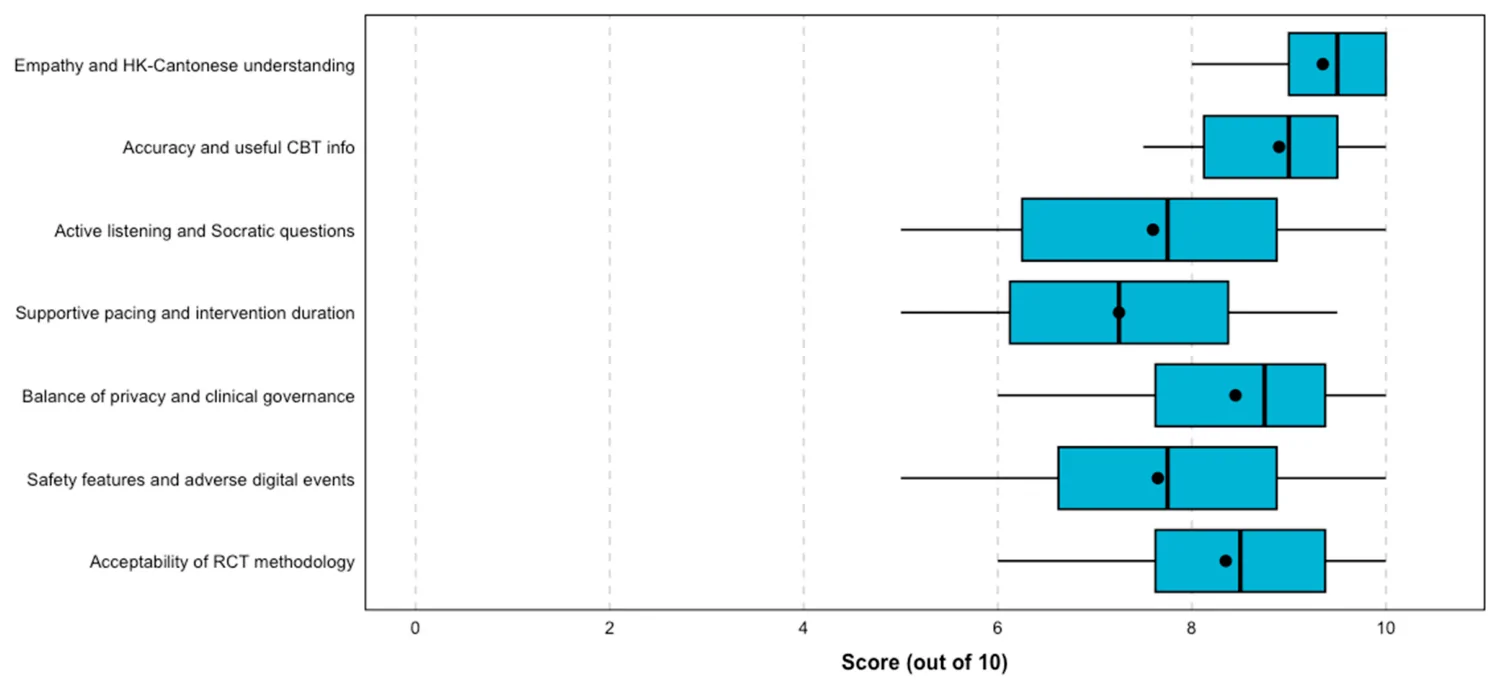
Boxplot distribution of descriptive stakeholder ratings on the seven feedback domains for CARES-MDD (10-point visual analogue scale; *n* = 18). Black dots indicate means. Abbreviations: CARES-MDD, Conversational Artificial-intelligence for REducing Symptoms and REsponse for Major Depressive Disorder; CBT, cognitive behavioral therapy; HK, Hong Kong; RCT, randomized controlled trial.

Following the Phase 1 needs assessment, 10 LEEs (mean age 22.9 ± 2.9 years; 50% female, 50% male) and eight clinical experts were invited to the initial workshop for co-design (Appendix A Table A1). They were then invited to test-run CARES-MDD to evaluate its acceptability, perceived safety, and feasibility of progression to a definitive trial of CARES-MDD. Only the LEEs had the pre- and post-observations adapting the outcome assessors. Descriptive ratings from the study-specific seven feedback domains are summarized in Figure 3.

Within this small stakeholder reference group, participants provided generally favorable ratings across all CARES-MDD feedback domains. Empathy and HK-Cantonese understanding, Accuracy and useful CBT info, and Balance of privacy and clinical governance achieved the highest median scores. These findings indicate positive preliminary stakeholder perceptions of the prototype’s culturally resonant communication, perceived usefulness and acceptability, and value of clinician oversight.Ratings regarding acceptability of a future randomized evaluation suggested that participants considered the proposed trial approach broadly acceptable. However, no prespecified feasibility criteria or formal consensus threshold was used; therefore, these ratings should not be interpreted as demonstrating feasibility of a future trial. Safety features and adverse digital events alongside active listening and Socratic questions showed wide interquartile ranges, suggesting variation in stakeholder perceptions, while others found automated overrides abrupt and that Socratic questioning could be demanding during distress. Finally, Supportive pacing and intervention duration yielded the lowest median score, capturing challenges foreseeable if it is positioned as an open-ended, indefinite intervention.The descriptive observations of the CARES-MDD test-run were summaraised in Table A5. Additionally, Table 3 thematically organizes the qualitative feedback underlying these quantitative evaluations.

Among the positive experiences, LEEs valued CARES-MDD as a private “tree hole” for emotional disclosure and perceived it as more private than commercial applications because it was not perceived to use disclosed content for targeted advertising that would pass the information to vendors. The use of natural Hong Kong-style Cantonese was viewed as familiar, emotionally accessible, and appropriate to local expressions. CBT-informed content was generally regarded as clinically appropriate and distinguishable from generic emotional support after several active exchanges. To ensure privacy, participants advocated for using pseudonyms to prevent data misattribution. However, they simultaneously supported backend clinician oversight, noting that human supervision of their conversations provided necessary reassurance against potential AI unreliability. Rather than limitations, their sharing of negative experience establish parameters for future trial methodology. Specifically, the cognitive demands of a text-based modality indicate that the intervention is optimally targeted toward a specific demographic: namely, younger, digitally native populations with a higher baseline tolerance for typing. Furthermore, the eventual onset of digital fatigue suggests that CARES-MDD is most viable as a time-limited intervention instead of an open-ended, indefinite intervention. Technologically, the LLM occasionally exhibited inconsistent longitudinal memory, failing to seamlessly recall contextual details from very early sessions, a stark contrast to human psychotherapists. Automated safety overrides were sometimes perceived as abrupt, particularly when triggered by non-suicidal colloquial exclamations such as “死啦” (“oh no”). Some participants expressed residual concern about possible AI hallucinations, despite not reporting such experiences during the study.

## 4. Discussion

This study reported a mixed-methods study: first, to characterize the online digital behavior, usage of generic AI chatbots for emotional concerns, its barriers, and needs assessment of desired AI mental health chatbot specifications; second, to co-design and evaluate the iterative test-run of the CARES-MDD among experts living with MDD. By bridging clinical needs-assessment data (Phase 1), we identified (1) barriers of self-stigma and fear of human judgement for sharing emotional concerns in add-on support within the existing psychiatric care framework; (2) high technological readiness with generic AI chatbots for emotional concerns; and (3) the desired features for development of an AI mental health chatbot for this specific clinical demographic. Phase 2 reported an iterative expert co-design process of CARES-MDD involving LEEs and a clinical expert reference group. They prioritized and shared their positive experience regarding CARES-MDD’s features of local style communication, CBT-curated RAG instead of generic content, and the balance of privacy and safety monitoring. Rather than limitations, their sharing of their negative experience regarding LLM-related risks and intervention pacing highlights key areas requiring reference parameters for future trial methodology.

Among the subgroup of Phase 1 respondents who provided voluntary open-ended comments, recurring categories included privacy concerns, fear of negative judgement, and concern about burdening others. These findings provide contextual insight into potential barriers to emotional disclosure even among this vulnerable cohort already attending psychiatric outpatient clinics [26]. These barriers may reduce willingness to seek help even when clinical need is present, particularly when the clinical population fears that their emotional disclosures will be misunderstood, shared beyond their control, or imposed on others [27,28]. A generative AI chatbot has the potential as an add-on micro-intervention to foreground immediate accessibility without burdening people, employing a conversational register that approximates support interaction rather than formal assessments.

It was further supported by the findings that most outpatient attendees had high technological readiness, with widespread surface acquaintance with generative AI chatbots. Users who have interacted briefly with commercial chatbots may hold implicit expectations derived from those platforms (rapid factual responses, task completion, minimal affect) that are poorly matched to the longitudinal, reflective, emotionally containing functions that a therapeutic chatbot must serve [24]. Among the 42 respondents who commented on desired features, recurring categories included immediate availability, a private and non-judgmental conversational space, natural local style communication, and practical psychological guidance. These comments informed the desired priorities of the subsequent process design priorities for the subsequent co-design process but should not be interpreted as population-level preferences.

The Phase 2 evaluation indicated generally favorable perceptions across the seven CARES-MDD feedback domains. Empathy and local style understanding, the accuracy and usefulness of CBT-informed content, and balance of privacy and safety received the highest ratings. Participants valued CARES-MDD as a private “tree hole” for emotional disclosure. The preference for unique identifiers or pseudonyms further illustrates the importance of maintaining confidence that individual disclosures will not be confused with those of another user. Participants appreciated that CARES-MDD responded in colloquial Cantonese rather than formal written Chinese, and several noted that this linguistic calibration created a sense of relational proximity uncommon in health-technology products [29,30].

Participants did not frame privacy as the absence of clinical oversight. Rather, they supported proportionate human oversight when data governance arrangements were transparent and sensitive personal details were protected. This finding is important for the design of clinically oriented conversational AI: trust may depend on clearly communicating what is monitored, who can access information, what will be documented, and the circumstances under which confidentiality may be overridden because of safety concerns.

CBT-informed content was perceived as more useful than generic reassurance when interactions progressed beyond short exchanges. Participants and experts considered the chatbot more helpful when it could validate distress, clarify the immediate concern, and only subsequently introduce structured reflection or cognitive restructuring. This supports a staged conversational design: initial empathic listening and emotional validation should precede problem solving or Socratic questioning. The interactions were perceived as more useful after several active exchanges, particularly when a session involved at least several active exchanges. This observation addresses a methodological challenge in DHI research: operationalizing intervention “dosage” outside of traditional psychiatric frameworks. Unlike conventional psychotherapy, which relies on structured 50 min appointments, unguided digital tools face tremendous variability in user engagement duration. Because meta-analyses indicate that an effective clinical dosage for digital interventions ranges from 30 to 60 min per week [31], future RCTs can formally define an “active digital session” as several active exchanges.

Several LEEs also reported that abrupt safety override responses, while appropriate from a risk-management standpoint, interrupted the conversational flow at moments when continued empathic engagement may have been more therapeutically productive. Clinical professionals specifically noted that, while algorithmic safety gates are non-negotiable from a duty-of-care standpoint, their current implementation in generative AI systems typically operates as a binary interrupt—abruptly redirecting conversation to crisis resources—rather than as a graduated, therapeutically calibrated transition. In human clinical practice, the decision to escalate, contain, or stay present with distress is a moment-to-moment clinical judgement informed by relationship history, non-verbal cues, and dynamic risk appraisal.

A central question explored during the co-design interviews was how clinical users conceptualize the identity of CARES-MDD. Instead, users perceived the CARES-MDD prototype as a portable, on-demand conversational support and skills-coaching tool during unassisted hours (e.g., late nights or between outpatient visits). Multiple LEEs affirmed their willingness to recommend CARES-MDD to peers who ‘aren’t ready for traditional therapy yet.’ By allowing distressed individuals to practice cognitive restructuring and emotional articulation in an unobserved, safe environment, the conversational agent serves as an accessible entry point that may lower psychological barriers and build readiness for future formal human psychiatric care.

The integration of generative AI into psychiatric populations raises broader ethical and clinical considerations for the field, particularly regarding unhealthy dependence, hallucination risks, and longitudinal memory constraints [32,33,34]. Their qualitative feedback highlighted a known vulnerability inherent to current LLMs [35]. LLM models unable to dynamically contextualize the psychotherapeutic clinical formulation of the engaging client unlike a human therapist may be better served as a complementary tool for “augmenting” pre-existing therapeutic alliance with patients, rather than serving as the therapist alliance in a standalone role [36]. In the emerging era of AI, we should be more cautious in the role of generative AI chatbots and monitor possible safety issues and adverse digital events. Although the LEEs did not experience these, they mentioned the risk of AI hallucinations and psychosis that they overheard. The LLM risks identified in the process are summarized in the subsequent Table 4, aligning with the guideline for a generative AI chatbot architecture ready for future trials [37].

Participants’ comments regarding long-term engagement should be interpreted as design considerations for future evaluation rather than as evidence of an optimal intervention duration. Participants anticipated digital fatigue if CARES-MDD was available as an open-ended or indefinite intervention. In light of attrition commonly reported in unguided digital mental health interventions, a time-limited, on-demand format may offer a pragmatic starting point for prospective feasibility testing. An approximately 8-week intervention window could allow participants sufficient opportunity to engage with CBT-informed self-management content while limiting prolonged exposure and the potential for overreliance on the conversational agent [38,39]. To mitigate the digital fatigue issue, further RCT trials could integrate the ecological momentary assessment technique, which has shown evidence of boosting adherence through self-monitoring and reminder effects [40,41].

Participants generally considered randomization, including allocation to a psychoeducation-only active digital control, to be fair and acceptable. Future trial design should minimize participant burden, particularly for providing appropriate reimbursement, online modes for assessments; and include follow-up sufficient to examine engagement, acceptability, safety-related events, and longer-term outcomes. While this formative study provided evidence on the architectural feasibility, cultural acceptability, and safety governance boundaries of CARES-MDD, it was not designed to evaluate randomized clinical efficacy or comparative attrition. Therefore, rather than demonstrating clinical superiority, these findings provide the critical structural parameters, such as the intervention dosage, the system and clinical measures to counteract the potential LLM risks, e.g., pre-consented clinical escalation pathways, required to safely initiate a definitive RCT.

### Strengths, Limitations, and Future Clinical Directions

A strength of this investigation is its mixed-methods methodology, uniting a relatively large clinical sample (*N* = 511) with more in-depth mixed-methods evaluation of LEEs throughout the development lifecycle, and CARES-MDD shows acceptable ecological and cultural validity. However, several limitations must be acknowledged. First, the pre-to-post observations from the uncontrolled beta-testing phase are strictly descriptive; they could not deduce therapeutic efficacy, and further clinical trial is needed. Second, it was a convenience sample drawn from a single professional and community network, which limits the generalizability. Without a screening log, we cannot determine response rates or compare non-participants. Due to potential selection bias from voluntary enrollment, these findings should be viewed as formative data rather than population-representative prevalence estimates. Nevertheless, given the uncompensated time commitments required for these phases of co-development, leveraging a network with pre-established clinical rapport was essential to maintain experts and accurately capture user-driven needs within existing resource constraints. Third, CARES-MDD intentionally avoids highly experimental technological features. This design choice was driven by the co-design participants, who prioritized clinical reliability and evidence-based safety over technological novelty, which might lead to “blackbox” concerns. While this limits the platform’s technological boundary pushing, it ensures the intervention maintains the rigorous clinical safety required for vulnerable psychiatric populations. Fourth, the Phase 1 needs assessment included adolescents aged 14–19 years, whereas the Phase 2 co-design group comprised adults aged 18 years or older with confirmed MDD. Adult LEE feedback may inform early prototype design but cannot establish acceptability, usability, safety, or clinical relevance for minors. This distinction is particularly important because some younger participants may have restricted smartphone access during school hours and because generative AI deployment among minors requires additional safeguards relating to assent, parental consent, privacy, crisis escalation, and school-context implementation. Future research should undertake adolescent-specific co-design and a prospectively approved feasibility study before extending CARES-MDD to people aged under 18 years.

Future work in the development of generative AI chatbots for mental health purposes must prioritize three interrelated agendas to address the universal challenges. Technically, the field should move beyond aligning global clinical guidelines and standardizing protocols for LLM applications across the disciplines [42,43]. Clinically, the next appropriate evaluative step involves prospective designs, such as full-scale RCTs, to systematically examine the clinical effectiveness. Digital adverse events should be continually assessed in future trials. Conceptually, the pronounced help-seeking barriers and high self-stigma identified in Phase 1 motivate a critical agenda for comparative research: investigating whether initial AI-mediated emotional disclosure can serve as a transitional bridge within a stepped-care pathway, lowering psychological barriers and facilitating subsequent engagement with human-delivered psychiatric care [44]. Addressing these universal challenges will be paramount for integrating generative AI as a safe, regulated, and continuously audited adjunct to global mental health services [11].

## 5. Conclusions

In conclusion, the broad needs assessment demonstrates that, while the use of generic AI chatbots for emotional support is already prevalent within a heterogeneous cohort of young people attending psychiatric outpatient services in Hong Kong, barriers—namely fear of judgment and burdening others—continue to impede traditional help-seeking behaviors within the traditional psychiatric care system. The needs assessment was not restricted to people with MDD; rather, it reflected the diagnostic complexity and comorbidity encountered in routine child and adolescent psychiatric practice. This underscores the necessity that generative AI chatbots with specified features are needed for individuals living with MDD, even among those already engaged in psychiatric care. By utilizing a needs-driven participatory framework with LEEs, a CBT-grounded generative AI chatbot for MDD has been co-developed, with mixed-methods findings supporting its feasibility to end-users and clinical experts. The findings provide preliminary, clinically grounded design parameters and feasibility information for subsequent evaluation. A future trial should compare CARES-MDD plus usual care with an appropriate active digital control plus usual care, incorporate predefined safety and adverse-event monitoring, and evaluate engagement, long-term safety, and clinical outcomes. This mixed-methods study does not establish the efficacy, safety, or therapeutic equivalence of CARES-MDD; these questions require subsequent prospective controlled trials.

## Figures and Tables

**Table 1:** Study-specific seven-domain feedback rubric for CARES-MDD for their acceptability, perception of safety and their opinions on a future trial design.

Evaluation Domain	Assessment Question	Clinical Rationale
Empathy and HK-Cantonese understanding	Cantonese understanding Did CARES-MDD express empathy and validate negative emotions naturally using locally acceptable language and tone?	An empathetic, localized understanding of the patients’ emotions reduces the barrier to disclosure and builds therapeutic alliance.
Accuracy and useful CBT info	Did CARES-MDD provide accurate CBT information and responses rather than providing superficial advice?	Skilled agents must address accurate CBT frameworks (e.g., cognitive restructuring); aids recovery rather than just surface-level symptoms.
Active listening and Socratic questions	Did CARES-MDD use active listening, followed by appropriately timed Socratic questioning?	Appropriate pacing of validation before Socratic questioning promotes self-exploration without feeling demanding.
Supportive pacing and intervention duration	Based on your experience with CARES-MDD’s pacing and technical glitches, what are your recommendations regarding the optimal intervention duration to sustain engagement without causing digital fatigue?	Identifying the technical barriers that hinder interaction and identifying the onset of digital fatigue to determine an acceptable duration of “dosage” for a future definitive RCT while balancing retention.
Balance of privacy and clinical governance	Did CARES-MDD balance the clinical monitoring with clinician oversight with the user’s need for respect of privacy?	Establishing transparent privacy boundaries while maintaining necessary clinical monitoring is essential for ethical governance, ensuring users feel secure enough to disclose without fearing unwarranted data integration.
Safety features and adverse digital events	Did CARES-MDD’s automated safety interface provide a necessary safety net without causing disruptive frustration, and did the interaction induce feelings of being dismissed by a machine?	Evaluating the trade-off between strict automated risk mitigation and therapeutic flow while proactively identifying potential iatrogenic harm, which is a safety prerequisite for ethical governance of a future RCT.
Acceptability of RCT methodology	Will persons living with MDD be willing to be randomized (including a psychoeducation control arm) and be comfortable with the proposed evaluation project timeline?	Validates the feasibility of a proposed RCT design, ensuring the recruitment strategy, assessment burden, and control comparators are acceptable to the target clinical demographic.

Abbreviations: CARES-MDD, Conversational Artificial-intelligence for REducing Symptoms and REsponse for Major Depressive Disorder; CBT, cognitive behavioral therapy; HK, Hong Kong; MDD, major depressive disorder; RCT, randomized controlled trial.

**Table 2:** Phase 1 clinical needs-assessment findings. *Note*. SD = standard deviation; PHQ-9 = Patient Health Questionnaire-9. Note: Phase 1 depression groups were defined using PHQ-9 symptom scores and do not represent DSM-based diagnoses of MDD. MDD diagnosis was confirmed only for the Phase 2 lived-experience expert group. ^a^ Welch’s *t*(396.1) = −6.48, *p* < 0.001, Hedges’ *g* = −0.60. ^b^ Welch’s *t*(355.3) = −7.42, *p* < 0.001, Hedges’ *g* = −0.71.

Variable	Result
Average Time per day spent online	296.3 min/day
Barriers to sharing emotional difficulties within traditional psychiatric system	Privacy concerns, Fear of judgment (negative consequences), Burdening others (causing trouble)
Help-Seeking Behaviors towards traditional support systems (Overall Score, max 28)	14.22 ± 5.26
Help-Seeking towards traditional support systems, mean difference (95% CI) ^a^: Depressed vs. Non-depressed	Depressed: 12.37|Non-depressed: 15.40, −3.03 [−3.95, −2.11] (*p* < 0.001)
Self-Stigma (Overall Score, max 24, higher = lower stigma)	14.05 ± 4.79
Self-Stigma mean difference (95% CI) ^b^: Depressed vs. Non-depressed	Depressed: 12.10|Non-depressed: 15.29, −3.20 [−4.05, −2.35] (*p* < 0.001)
Prevalence of generic AI chatbot Adoption (95% CI)	66.2% [61.9–70.1%]
Average AI chatbot Time Spent, median (IQR); mean ± SD (Entire Sample)	20 (0–120); 56.9 ± 87.7 min/day
Average AI chatbot Time Spent, median (IQR); mean ± SD (Active Users Only)	60 (23–122); 85.9 ± 95.5 min/day
Frequency of AI chatbot Interactions (Active Users)	<10 total conversations: 56.4% 10–50 conversations: 19.8% Several times a week: 13.9% Up to 2 h daily: 5.1% 2–5 h daily: 4.9%

**Table 3:** Users’ experiences of good and bad points for the CARES-MDD chatbot.

Mention Level and Category	Positive Points	Negative Points
Most mentioned		
App-based modality and usability	Functions effectively as a private “treehole” where users feel comfortable sharing anything; perceived as much safer than commercial mental health apps due to the absence of targeted advertising based on disclosed content. Its use of natural, Hong Kong-style Cantonese was perceived as familiar and emotionally accessible, and its understanding of local expressions was regarded as appropriate.	Several participants suggested that the intervention might be less suitable for older users or individuals with limited motivation, concentration, or comfort with typing, given the need for regular text input. However, the text-based format allowed for the use of CARES-MDD without being overheard and access in settings where speaking with another person would be difficult.
Therapeutic features (CBT-informed psychoeducation and self-management prompts)	Experts rated the CBT information as clinically appropriate; the chatbot maintains a structured, supportive tone that differentiates itself from generic emotional venting. The system was perceived as more useful, particularly after several active exchanges.	Requires at least 5 active interactions to move beyond superficial advice. Socratic questioning can feel demanding if the AI misjudges the user’s readiness, occasionally sounding “fake”. Struggles to switch back to a simple venting mode when structured CBT is no longer desired.
Frequently mentioned		
Study design and intervention pacing	The conversational format allowed participants to engage at their own pace and return to the system when support was needed. The extended intervention period provided an opportunity to observe engagement across various stages of use.	Participants identified digital fatigue resulting from unlimited use. Several participants mentioned technical limitations included inconsistent recall of contextual details from earlier sessions, which contrasted with the continuity expected from a human therapist.
Moderately mentioned		
Safety features and adverse digital events	Users are comfortable with data syncing to clinical logs provided that distinct pseudonyms (different login names) are used to prevent data mixing. Users value that specific, intimate details are shielded from their primary treating psychiatrists. Some underlying anxiety remains regarding potential AI hallucinations based on external news reports, though not directly experienced.	The automated safety override feels abrupt and can be triggered prematurely by false-positive slang exclamations during normal emotional venting (e.g., ‘死啦’/‘Oh no’). Follow-up and systematic and clinical safety monitoring were considered necessary.
Less mentioned		
Acceptability of future randomized evaluation	Participants viewed an active-control randomized controlled trial as acceptable and potentially valuable for distinguishing intervention effects from expectancy effects. They considered the proposed outcome measures broadly appropriate and supported longer-term follow-up.	Participants noted the assessments are necessary but time-consuming and recommended adequate compensation for participants. Active control is considered as more “fair” to those joining RCT as they would expect to be assigned to a “helpful” group if participating.

Abbreviations: AI, artificial intelligence; CARES-MDD, Conversational Artificial-intelligence for REducing Symptoms and REmission for Major Depressive Disorder; CBT, cognitive behavioral therapy; RCT, randomized controlled trial.

**Table 4:** Stakeholder-informed safeguards and clinical oversight considerations for potential LLM-related risks in CARES-MDD.

Identified LLM Vulnerability	System-Level Architectural Safeguard	Human-in-the-Loop (HITL) and Clinical Oversight
AI Hallucinations and Reinforcement of Harmful Viewpoints	Retrieval-augmented generation responses are constrained by clinician-reviewed CBT-informed knowledge resources to mitigate the chance of extrapolating “hallucinatory content” beyond validated, evidence-based frameworks.	Samples of transcripts will be reviewed weekly for fidelity to intended content and potential safety concerns.
“Context Rot” (Inconsistent Longitudinal Memory)	The system is explicitly designed for brief, bounded interactions rather than continuous, long-term narrative therapy, thereby minimizing the model’s reliance on deep historical recall.	Users are informed during onboarding that the AI is a present-moment skills coach, managing expectations regarding the AI’s long-term memory capabilities.
Iatrogenic Risk of Abrupt Automated Crisis Overrides and Imminent Risk	Supportive crisis-signposting interface: the system triggers a supportive safety interface offering localized crisis hotlines alongside the dialogue, avoiding abrupt conversational termination.	Consent is obtained during onboarding stipulating that, despite using pseudonyms, the research team is authorized to contact the patient for contingency according to the safety protocol.
Digital Fatigue and Unhealthy Dependency	The intervention protocol will be structurally bounded to a maximum active period to prevent chatbot use from displacing usual psychiatric care.	Evaluates the retention and engagement time of active sessions. Integrates ecological momentary assessment (EMA) techniques that have shown evidence of boosting adherence through self-monitoring and reminder effects.

Abbreviations: AI, artificial intelligence; CARES-MDD, Conversational Artificial-intelligence for REducing Symptoms and REmission for Major Depressive Disorder; CBT, cognitive behavioral therapy; EMA, ecological momentary assessment; HITL, human-in-the-loop; LLM, large language model.

## Data Availability

To protect participant anonymity and comply with ethics board confidentiality agreements, raw datasets containing identifying information (e.g., specific school names and personal open-ended responses) cannot be made public. The data is available from the corresponding author upon reasonable request.

## Abbreviations

The following abbreviations are used in this manuscript:

AI	Artificial Intelligence
CARES-MDD	Conversational Artificial-intelligence for REducing Symptoms and REsponse for Major Depressive Disorder
CBT	Cognitive Behavioral Therapy
DHIs	Digital Health Interventions
DSM-5	Diagnostic and Statistical Manual of Mental Disorders, Fifth Edition
Gen-AI	Generative Artificial Intelligence
HITL	Human-in-the-loop
HK	Hong Kong
HS	Help-Seeking
KAMHS	Knowledge and Attitudes to Mental Health Scales
LEE	Lived-Experience Experts
LLM	Large Language Model
MDD	Major Depressive Disorder
PHQ-9	9-item Patient Health Questionnaire
RCT	Randomized Controlled Trial
SD	Standard Deviation
SS	Self-Stigma
YPAR	Youth Participatory Action Research

## Appendix A

**Table A1:** Prioritized co-design implementation ideas for the CARES-MDD intervention. *Note*. Priority rank reflects the order in which implementation ideas were prioritized within each co-design category. Phase 1 findings were presented as design prompts, and stakeholder suggestions were prioritized and translated into implementation actions during iterative co-design and supervised test-runs.

Evaluation Domain (Co-Design Category)	Priority Rank	Stakeholder Input and Translation	Implementation Idea
Empathy and HK-Cantonese Understanding	1	Phase 1 findings on disclosure barriers were presented as design prompts; LEEs prioritized local language and emotional validation.	Integrate natural Hong Kong-style Cantonese syntax and culturally appropriate conversational sensibilities to lower disclosure barriers.
	2	LEEs requested validation before problem solving during test-runs.	Program the agent to explicitly validate negative emotions and distress before attempting to initiate problem-solving frameworks.
	3	LEEs identified repetitive or artificial responses during co-design discussions and supervised test-runs.	Diversify empathetic phrasing to prevent repetitive, artificial responses during longitudinal interactions.
Accuracy and Useful CBT Info (Therapeutic Fidelity)	1	Phase 1 findings for practical psychological guidance were reviewed with clinical professionals and translated into modular CBT content.	Architect the system using modular knowledge banks (e.g., an eight-knowledge-bank structure) to deliver structured CBT.
	2	LEEs and clinicians requested structured guidance rather than generic reassurance.	Guide users systematically through cognitive restructuring exercises rather than offering superficial advice.
	3	Clinical professionals prioritized cognitive-pattern support without diagnostic inference.	Implement algorithmic detection of cognitive distortions (e.g., highlighting absolute words such as “always” or “must”) based on user input.
Active Listening and Socratic Questions	1	LEEs and clinicians reported that questioning could feel demanding during distress.	Calibrate the pacing of Socratic questioning to evaluate user readiness, ensuring prompts do not feel demanding during acute distress.
	2	LEEs prioritized supportive listening before structured guidance.	Provide supportive listening before transitioning into structured clinical guidance.
	3	LEEs requested an option to return to unstructured emotional expression.	Develop a seamless conversational mechanism allowing users to easily revert to an unstructured “treehole” venting mode when structured CBT is no longer desired.
Supportive Pacing and Intervention Duration	1	Test-run feedback highlighted digital fatigue.	Bound the testing window to an on-demand use instead of unlimited use to observe any adverse digital events and prevent digital fatigue.
	2	Clinical professionals highlighted depressive amotivation and psychomotor slowing.	Accommodate severe depressive amotivation and psychomotor slowing by removing immediate session timeouts, allowing asynchronous engagement.
	3	LEEs identified typing burden as a usability concern.	Optimize the interface to reduce typing burden where clinically appropriate, recognizing the cognitive fatigue associated with major depressive disorder.
Balance of Privacy and Clinical Governance	1	Phase 1 findings identified concerns about negative judgement and disclosure within existing care relationships; LEEs recommended pseudonyms or separate user identifiers to reduce data misattribution.	Assign distinct pseudonyms or unique login identifiers to prevent data misattribution and ensure anonymity from primary treating teams.
	2	LEEs and clinicians jointly discussed privacy boundaries and escalation.	Shield intimate conversational disclosures from the patient’s primary treating psychiatrist while allowing PI to inform case psychiatrists if worsening symptoms.
	3	LEEs and clinicians recommended transparent clinician oversight from onboarding.	Provide transparent, upfront psychoeducation regarding backend clinician oversight to reassure users about AI reliability and data governance.
Safety Features and Adverse Digital Events	1	LEEs and clinicians prioritized crisis signposting and safety overrides.	Implement automated crisis contingency messaging and safety overrides when crisis words are detected.
	2	LEEs reported that colloquial expressions could trigger abrupt alerts.	The contingent interface can appear, but user can choose to close this interface afterwards for troubleshooting the colloquial venting exclamations.
	3	Clinical professionals mentioned the events of hallucinations from recent literature.	Keep clinician oversight to supervise interactions and safeguard against potential large language model hallucinations.
Acceptability of RCT Methodology	1	Phase 1 findings for “treehole” and importance of “privacy”; LEEs valued privacy, secure hosting.	Specify secure university-hosted deployment and transparent data-governance arrangements.
	2	LEEs identified assessment burden as a concern.	Give reimbursement for time and allow online assessment (e.g., via zoom) related to psychometric outcome assessments to minimize participant time burden and during the follow-up period.
	3	LEEs felt an active comparator as fairer and or else could be disappointed.	Allow the other arm also to use active digital control, or else sense unfairness for the control arm

Abbreviations: AI, artificial intelligence; CBT, cognitive behavioral therapy; HK, Hong Kong; LEEs, lived-experience experts; PI, principal investigator; RCT, randomized controlled trial.

**Table A2:** CARES-MDD system identity, intended use and technical configuration.

System Name	CARES-MDD (Conversational Artificial-Intelligence for REducing Symptoms and REsponse for Major Depressive Disorder)
Intended users	People attending psychiatric clinics in Hong Kong, currently using a smartphone that has internet access, and who speak Cantonese Chinese
Intended use	An adjunctive therapeutic tool delivering cognitive behavioral therapy (CBT) exercises (e.g., cognitive restructuring), empathetic validation, and on-demand emotional support between formal psychiatric outpatient appointments
Clinical boundaries	Not designed for diagnosis, emergency care, medication advice, crisis management, or replacement of standard treatment
Language and register	Hong Kong-style Cantonese
Underlying model	Requested on demand
Deployment setting	API hosted/processed in the server hosted within the university; chat content was not transferred to an external model provider
Generation approach	Structured system prompting RAG, with fine-tuning; lexical crisis screening triggers a predefined safety override
Sources of the RAG	CBT-informed materials developed/curated by psychiatrists and clinical psychologists and reviewed by the clinical team and expert-curated therapeutic dialogues developed by a multidisciplinary psychiatric team
Memory/context configuration	Cross-session information retained during active use
Safety mechanism	Real-time lexical screening for predefined high-risk crisis language; positive triggers suspend routine chatbot content and display a static emergency interface with 24 h crisis resources
Clinical-review arrangement	Authorized clinical team members conducted periodic weekly review of chatbot content and system responses for quality and safety refinement; this was not continuous real-time monitoring or emergency response
Participant data used for model training	No participant conversation data were used to train or fine-tune the underlying language model
Material updates during study	No material changes to the model, prompts, safety rules, memory configuration, knowledge resources, or user interface occurred during participant data collection

Abbreviations: API, application programming interface; CARES-MDD, Conversational Artificial-intelligence for REducing Symptoms and REmission for Major Depressive Disorder; CBT, cognitive behavioral therapy; RAG, retrieval-augmented generation.

**Table A3:** Safety screening and crisis-response pathway.

Stage	Implemented Process	Trigger/Category	User-Facing Response	Human Action	Important Limitation
Initial screening	Lexical-based	Direct suicidal language, such as “想自殺” (“want to commit suicide”), “要自殺” (“will commit suicide”), or “我想死” (“I want to die”); Death wishes or desire not to exist, such as “唔想生存” (“do not want to live”), “想消失” (“want to disappear”), “死咗就好” (“it would be better if I were dead”), or “希望自己唔再存在” (“wish I no longer existed”); Self-harm or injury-related language, such as “傷害自己” (“hurt myself”), “自殘” (“self-harm”) or statements indicating recent self-injury; Expressions involving possible methods or preparation, such as references to taking a large quantity of medication, obtaining means, preparing a suicide note, or making arrangements before death.	None (if negative)	No immediate human action for messages without a trigger.	Not a validated clinical risk assessment tool; highly susceptible to false positives from Cantonese colloquialisms (e.g., ‘死啦’/‘Oh no’ “死火喇” (“uh-oh” or “this is bad”), “去死啦” (“go away” or “go to hell”), “我死喇” (“I am in trouble” or “I am exhausted”). However, commonly understood expressions that are clearly unrelated to suicidal intent, e.g., “忙到死” (“extremely busy”), “攰到死” (“extremely tired”),份工玩死我” (“this job is exhausting me”), “功課多到想死” (“I have so much homework that I could die”), are not expected to trigger safety alerts because the system considers linguistic context rather than isolated death-related terms
Secondary check	Not applicable	Not applicable	Not applicable	Not applicable	No applicable
Safety override	Static emergency interface	Immediate upon detection of any initial screening keyword.	Static emergency message advising the user to seek urgent help and displaying 24 h crisis resources/direct-dial contact options	None in real time	May interrupt conversation abruptly
Escalation	Consent is obtained for emergency contact and liaising with their existing professional team in case of safety crisis scalation	Significant risk detected by screening authorized clinical member.	Participants are explicitly informed during onboarding and via the consent form that the tool is not an emergency service	Authorized clinical members conducted periodic weekly review of selected content and system responses for safety and quality refinement; this did not constitute continuous real-time monitoring or emergency response.	The research team needs to manually check user accessed services, contact a crisis resource, or receive a clinical response after viewing the emergency interface
Documentation	Automated event logging of all chat transcripts and triggered overrides	All user interactions and safety events.	Not applicable	Periodic weekly clinical review of transcripts by the research team.	Trigger-event logs and review records do not by themselves establish whether risk was present, whether a false-positive occurred, whether the user sought help, or whether harm was prevented

**Table A4:** This table describes the architecture of CARES-MDD. It is intended to clarify the system’s content sources, module boundaries, and routing approach.

Architecture Domain	CARES-MDD Specification, Content Governance, and Boundaries
Base model and deployment	CARES-MDD was implemented as a text-based conversational application using LLM. It is intended as an add-on support tool; it was not intended to diagnose MDD, prescribe treatment, provide emergency care, or autonomously manage suicide risk.
Knowledge-based design	Clinical content was organized into separate, function-specific knowledge banks. This separation was intended to prevent patient-facing conversations from retrieving therapist-only guidance, detailed suicide-assessment material, medication guidance, or research/governance documents. The knowledge-bank framework comprised CBT foundations (KB01), assessment and formulation (KB02), process-to-intervention rules (KB03), CBT intervention modules (KB04), Traditional Chinese worksheets (KB05), maintenance and relapse prevention (KB06), safety and escalation (KB07), and research/system evidence (KB08).
RAG source materials	CBT-informed materials developed/curated by psychiatrists and clinical psychologists and reviewed by the clinical team and expert-curated therapeutic dialogues developed by a multidisciplinary psychiatric team.
RAG inclusion criteria	Only short, topic-specific, patient-appropriate knowledge units (“topic cards”) were intended for retrieval. Each unit was to include a defined title, use indications, exclusions, brief patient-facing content, one-question-at-a-time prompts, completion criteria, escalation criteria, source/page metadata, and clinical review status. Detailed suicide-assessment material, diagnostic flowcharts, exposure/ERP hierarchies, trauma-trigger forms, and no-suicide contracts were excluded from unrestricted generation.
Assessment and formulation boundary	KB02 was designed to support a structured understanding of the user’s current concern through situation–thought–emotion–behavior links, goals, triggers, strengths, preferences, functional impact, and maintaining-process hypotheses.
Process-routing logic	The architecture proposed a process-to-intervention router (KB03) that identifies one primary maintaining-process hypothesis and selects one approved first-line module. Example mappings included behavioral activation for inactivity/anhedonia; thought monitoring and cognitive restructuring for negative automatic thoughts; emotion labeling and grounding for emotional overload; and structured problem solving for practical difficulties.
CBT module boundaries	The CBT-informed modules were intended to provide bounded psychoeducation and optional micro-exercises, including behavioral activation, automatic-thought monitoring, cognitive restructuring, grounding, relaxation, rumination management, problem solving, sleep/routine support, avoidance reduction, and interpersonal support. It was not authorized to provide medication advice or treatment recommendations outside approved content.
Traditional Chinese and cultural adaptation	Patient-facing exercises were selected and adapted for Traditional Chinese delivery, with a Hong Kong Cantonese conversational register. The intended design was to guide one brief exercise step at a time rather than deliver a full worksheet.
Data governance and privacy	The system was intended to use de-identified participant identifiers and restricted research-team access. Co-design feedback supported clear privacy boundaries, distinct identifiers/pseudonyms, and transparency regarding information that may be reviewed or shared.

Abbreviations: CARES-MDD, Conversational Artificial-intelligence for REducing Symptoms and REsponse for Major Depressive Disorder; CBT, cognitive behavioral therapy; ERP, exposure and response prevention; KB, knowledge bank; LLM, large language model; MDD, major depressive disorder; RAG, retrieval-augmented generation.

**Table A5:** Descriptive clinical-monitoring observations during CARES-MDD test-run. Note: HDRS-17 = 17-item Hamilton Depression Rating Scale; PHQ-9 = 9-item Patient Health Questionnaire; GAD-7 = 7-item Generalized Anxiety Disorder scale; SD = Standard Deviation. The maximum score for the Working Alliance Inventory is 60 and for Satisfaction with CARES-MDD is 7. These descriptive pre–post observations are reported for archival and trial-planning transparency only. Given the single-arm, uncontrolled design and small sample (*n* = 10 lived-experience experts), no inferential statistic was applied, as no such analysis could support a valid causal or correlational interpretation of change over the test-run period. These values must not be interpreted as evidence of clinical improvement, efficacy, or safety attributable to CARES-MDD.

	At the Beginning of the Test-Runs: Mean (SD)	At the End of the Test-Runs: Mean (SD)	Clinical Interpretation
HDRS-17	19.33 (6.67)	14.29 (6.37)	
PHQ-9	12.22 (4.49)	8.00 (3.92)	
GAD-7	10.89 (3.48)	8.86 (4.85)	
Working Alliance Inventory		43.1 out of 60 (11.9)	Moderate to Strong therapeutic alliance
Satisfaction with CARES-MDD	-	5.71 (0.76)/7	High overall satisfaction
System Usability Scale	-	72.14 (6.68)	Above-average usability (>70 threshold)
